# Clinical efficacy of minimally invasive transforaminal lumbar interbody fusion via bilateral channel for lumbar degenerative disease

**DOI:** 10.3389/fsurg.2026.1787824

**Published:** 2026-04-20

**Authors:** Ji-hui Zhang, Liang Yu, Jing-fei Xu, Jin-ming Han, Xu-yu Liao, Bo Chai, Liu-jun Zhao

**Affiliations:** 1 Department of Spinal Surgery, Ningbo No.6 Hospital, Ningbo, Zhejiang, China; 2 Ningbo Clinical Research Center for Orthopedics, Sports Medicine & Rehabilitation, Ningbo, Zhejiang, China; 3 Department of Medical Insurance, Ningbo No.6 Hospital, Ningbo, Zhejiang, China; 4 Department of Medical Record, Ningbo No.6 Hospital, Ningbo, Zhejiang, China

**Keywords:** bilateral channel, efficacy, interbody fusion, lumbar degenerative disease, minimally invasive surgery

## Abstract

**Objective:**

To evaluate the clinical efficacy of bilateral channel minimally invasive transforaminal lumbar interbody fusion (MIS-TLIF) in the management of lumbar degenerative diseases.

**Methods:**

A retrospective analysis was conducted of 68 patients diagnosed with lumbar degenerative diseases who underwent surgical intervention at Ningbo No.6 Hospital between April 2021 and February 2022. The patients were categorized into a traditional TLIF group (38 cases) and a bilateral channel MIS-TLIF group (30 cases). Comparative assessments were performed between the two groups in terms of surgical outcomes.

**Results:**

All surgical procedures were successfully performed and postoperative follow-up was maintained for (12.7 ± 1.7 months). Statistically significant differences were observed in operation time, intraoperative fluoroscopy frequency, intraoperative blood loss, postoperative drainage volume, and length of hospital stay between two groups. The VAS scores and ODI of the two groups measured at 7 days postoperatively and at the final follow-up were significantly lower than the preoperative values, with statistically significant differences. The fusion rates were 89.5% in the traditional group and 93.3% in the bilateral channel MIS-TLIF group, with no statistically significant differences.

**Conclusion:**

Bilateral channel MIS-TLIF is a feasible surgical procedure and it can reduce the surgical duration and radiation exposure associated with intraoperative fluoroscopy.

## 1 Introduction

Lumbar degenerative disease is caused by the degeneration of the lumbar spine and its surrounding structures. For patients with conservative failure and combined with lumbar instability, minimally invasive transforaminal lumbar interbody fusion (MIS-TLIF) is a conventional surgical method for the treatment of lumbar degenerative disease (1, 2).

Minimally invasive spine surgery has increasingly emerged as the future trajectory of spinal surgical interventions, with MIS-TLIF routinely employed for the management of lumbar degenerative disease. Compared to conventional open surgery, this technique has multiple advantages, including minimized paraspinal muscle trauma, reduced intraoperative blood loss, expedited postoperative recovery, and a shortened duration of hospitalization (3). However, in traditional MIS-TLIF procedures, screws are initially placed on the decompression side, and following confirmation of proper screw positioning, the contralateral side is instrumented. Alternatively, percutaneous screws can be inserted bilaterally after decompression and bone grafting. Both approaches contribute to a prolonged operative duration and increased radiation exposure due to fluoroscopy.

Therefore, the existing literature indicates that MIS-TLIF is associated with significantly prolonged operative durations and elevated radiation exposure due to fluoroscopic guidance (4–7). To address these concerns, a modified surgical approach incorporating bilateral channel MIS-TLIF has been developed to facilitate simultaneous screw placement via bilateral channels. This study aimed to evaluate the clinical efficacy of bilateral channel MIS-TLIF in the treatment of lumbar degenerative diseases.

## 2 Materials and methods

### 2.1 General characteristics

This retrospective cohort study retrospectively analyzed 68 patients diagnosed with lumbar degenerative disease who were treated at Ningbo No.6 Hospital between April 2021 and February 2022. Of all the patients, 38 patients underwent traditional TLIF (TLIF group, mean age: 55.9 ± 11.8 years), with 22 males and 16 females, comprising 27 cases of lumbar disc herniation, 9 cases of lumbar spinal stenosis, and 2 cases of lumbar spondylolisthesis, and 30 patients underwent bilateral channel MIS-TLIF (bilateral channel MIS-TLIF group; mean age: 60.2 ± 10.2 years), with 17 males and 13 females, including 20 cases of lumbar disc herniation, 8 cases of lumbar spinal stenosis, and 2 cases of lumbar spondylolisthesis. Both surgeries were performed by two doctors with more than five years of experience in TLIF and MIS-TLIF surgery.

The inclusion criteria were as follows: ① patients age between 18 and 80 years; ② patients with single segment degenerative diseases, including lumbar disc herniation with instability, lumbar spinal stenosis with instability and lumbar spondylolisthesis; ③ patients presented low back pain, accompanied by intermittent claudication and/or sciatica, neurological symptoms; ④ failure of conservative treatment for over three months.

The exclusion criteria were: ① the presence of mental or cognitive disorders; ② incomplete clinical records, lack of follow-up data, or loss to follow-up; ③ severe internal medical diseases; ④ a history of lumbar fracture, tumor, infection, or prior surgical intervention at the same spinal segment.

The study protocol was approved by the Institutional Ethics Committee of the Ningbo No.6 Hospital (CODE: 2024-37). Written informed consent was obtained from all study participants. All procedures were performed in accordance with the Declaration of Helsinki and relevant policies in China.

### 2.2 Surgical method

#### 2.2.1 Traditional TLIF group

Patients were in a prone position after general anesthesia. The incision was made in the middle direction along the spinous process in the segment to be operated on. Layer-by-layer dissection of tissues was performed to ensure adequate exposure of the lamina and facet joints. Bilateral pedicle screws (Medtronic Inc.) were inserted to achieve fixation and screw positioning was confirmed using C-arm. The facet joints, lamina and ligamentum flavum were excised to expose the disc space. After remove the annulus fibrosus and the nucleus pulposus, the cartilage plate above and below was removed. The intervertebral space was adequately filled with autologous bone fragments obtained from the excised lamina, followed by the insertion of one PEEK cage filled with bone fragments (Medtronic Inc.) into the intervertebral space. After connecting rods were secured, drainage was placed and incision was sutured.

#### 2.2.2 Bilateral channel MIS-TLIF group

**Figure 1:**
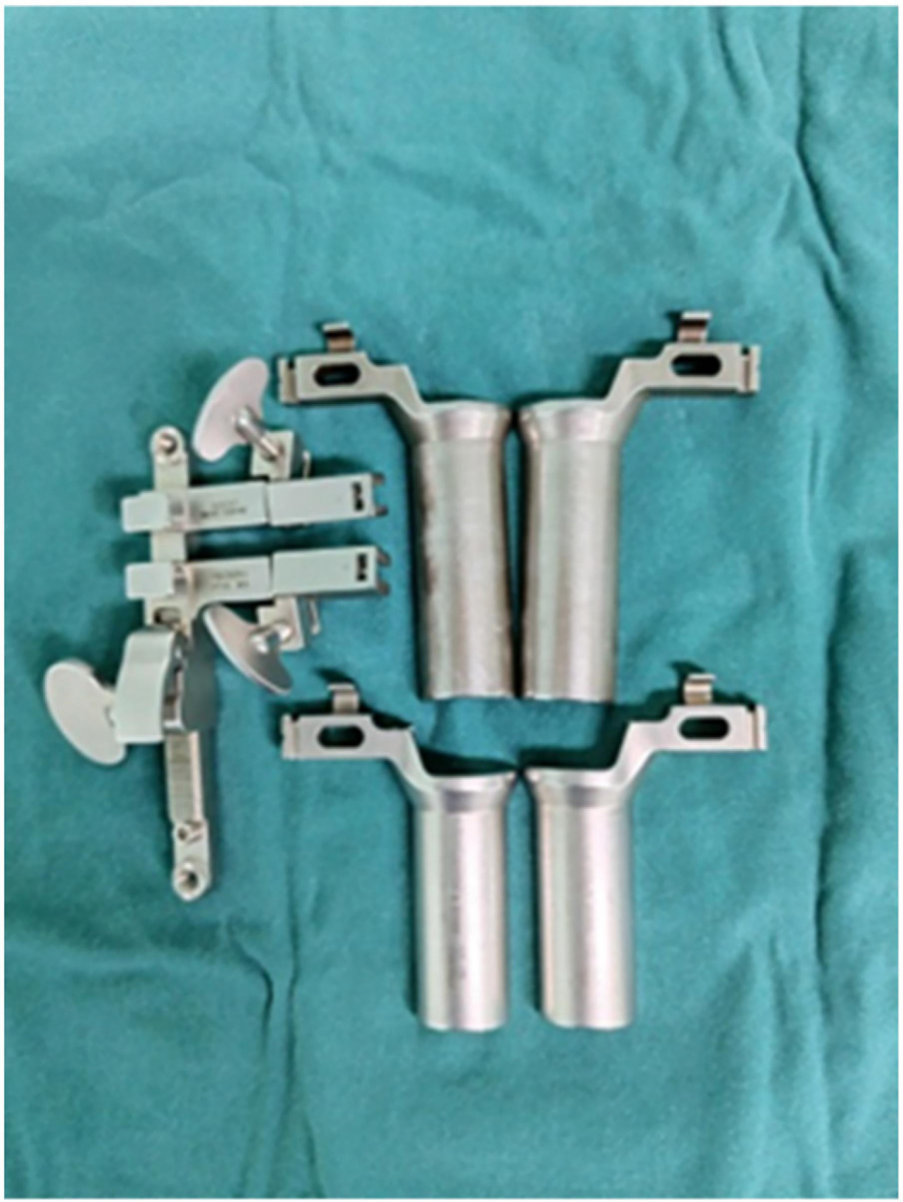
Five-piece set: working channel of quadrant system.

**Figure 2:**
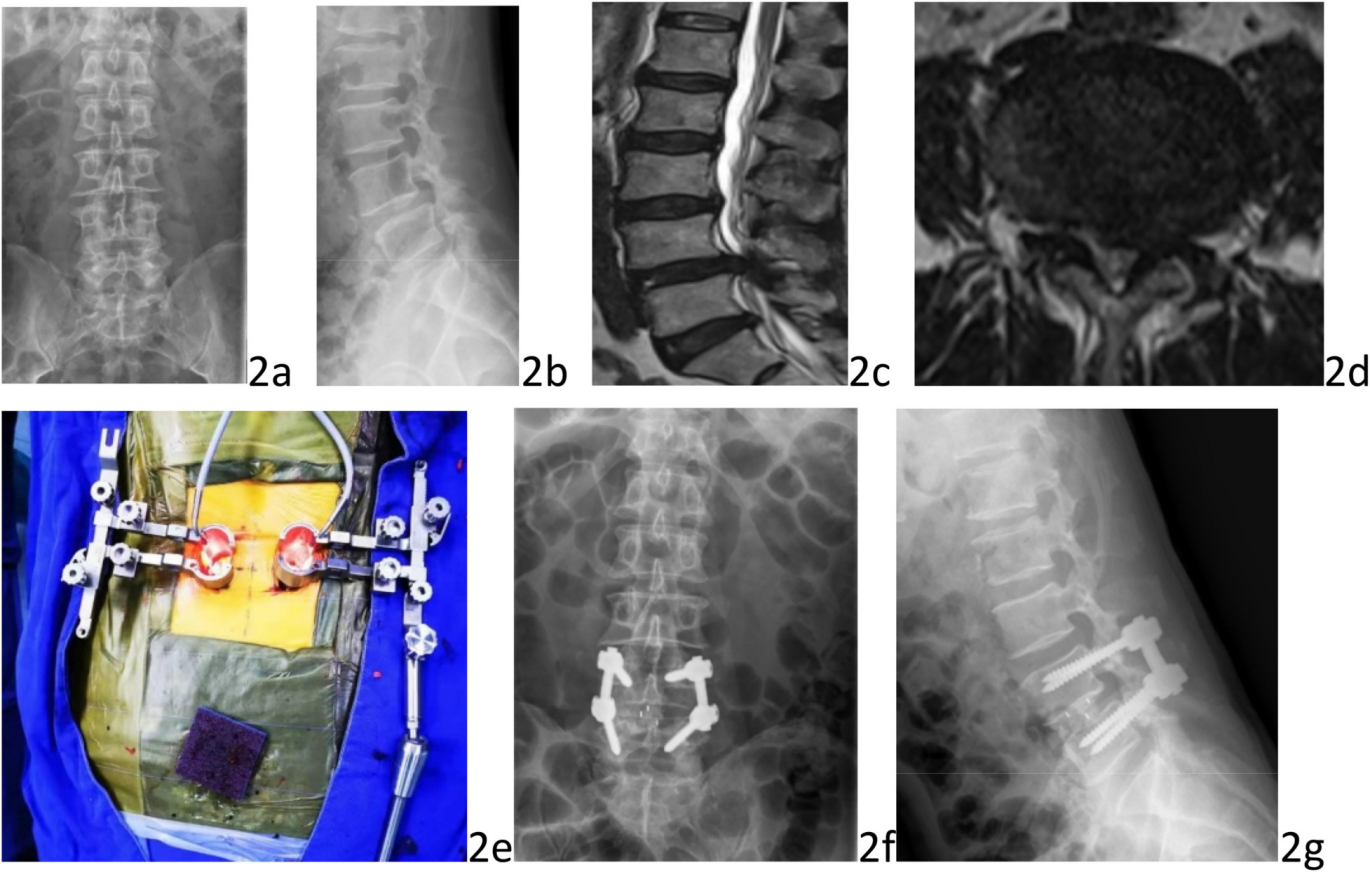
A 72-year-old male was diagnosed for lumbar disc herniation with preoperative, intraoperative, and postoperative images of bilateral channel MIS-TLIF. Preoperative anteroposterior and lateral radiographs showed lumbar instability **(a,b)**. Preoperative lumbar MRI showing L4/5 disc herniation with instability **(c,d)**. Intraoperative bilateral channel procedure **(e)**. Postoperative anteroposterior and lateral lumbar radiographs demonstrating satisfactory position of internal fixation **(f,g)**.

Patients were in a prone position after general anesthesia. The level of operation was confirmed under anteroposterior of the C-arm. A 4-cm longitudinal incision was made lateral to the midline on the decompression side, then extending through the skin and deep fascia. Blunt separate of the paraspinal muscles was performed. A quadrant system working channel was inserted, and then connect the cold light illuminator to expose the lamina and facet joints. The facet joints, lamina and ligamentum flavum were excised to expose the disc space. After remove the annulus fibrosus and the nucleus pulposus, the cartilage plate above and below was removed. The intervertebral space was adequately filled with autologous bone fragments obtained from the excised lamina, followed by the insertion of one PEEK cage filled with bone fragments (Medtronic Inc.) into the intervertebral space. When fusion almost finished, a separate 4-cm incision was made lateral to the midline on the non-decompression side by assistant, extending through the skin and deep fascia. The paraspinal muscles were bluntly separated and another quadrant system working channel (five-piece set, Figure 1) was inserted to expose the lateral edge of the facet joint. Bilateral pedicle screws (Medtronic Inc.) were inserted at the same time to achieve fixation and screw positioning was confirmed using C-arm. After connecting rods were secured, drainage was placed and incision was sutured (Figure 2).

### 2.3 Observation parameters

Operation duration, frequency of intraoperative fluoroscopy, intraoperative blood loss, postoperative drainage volume, length of hospital stay, fusion rate, and occurrence of complications were systematically recorded and analyzed for both groups. Additionally, the visual analog scale (VAS) scores and Oswestry Disability Index (ODI) were evaluated preoperatively, 7 days postoperatively, and during the final follow-up for both groups.

### 2.4 Statistical analysis

Statistical analyses were performed using SPSS 22.0. Measurement data are expressed as the mean ± standard deviation. Independent sample t-tests were used to compare the age, surgical parameters, follow-up duration, VAS scores, and ODI scores between the two groups. Paired t-tests were used to evaluate differences in the VAS and ODI scores across various follow-up time points within the same group. Categorical variables were analyzed with a Chi-squared test. Statistical significance was defined as *P* < 0.05.

## 3 Results

### 3.1 General information

All surgical procedures for the 68 patients were successfully performed by two chief physicians, with no major intraoperative complications reported. Additionally, as shown In Table 1, no statistically significant differences were identified in the baseline characteristics between the two groups.

**Table 1:** Information of traditional TLIF group and bilateral channel MIS-TLIF group.

Variables	Traditional (*n* = 38)	Bilateral channel (*n* = 30)	*P*
Gender (M/F)	22/16	17/13	0.919
Age (yr)	55.9 ± 11.8	60.2 ± 10.2	0.120
BMI (kg/m^2^)	28.2 ± 1.1	27.9 ± 1.1	0.305
Diagnosis			0.922
Lumbar disc herniation	27	20	
Lumbar spinal stenosis	9	8	
Lumbar spondylolisthesis	2	2	
Level of fusion			0.279
L3/4	2	5	
L4/5	25	16	
L5/S1	11	9	

### 3.2 Surgical outcomes

As shown in Table 2, the bilateral channel MIS-TLIF group exhibited a significantly shorter mean operative duration, fewer intraoperative fluoroscopic exposures, reduced intraoperative blood loss, decreased postoperative drainage volume, and a shorter length of hospital stay than the traditional TLIF group. All the differences were statistically significant (*P* < 0.05).

**Table 2:** Comparison of surgical outcomes between traditional TLIF group and bilateral channel MIS-TLIF group.

Variables	Traditional (*n* = 38)	Bilateral channel (*n* = 30)	*P*
Operation duration (min)	120.0 ± 10.7	89.2 ± 7.9	0.000
Frequency of intraoperative fluoroscopy	13.9 ± 1.6	9.5 ± 1.8	0.000
Intraoperative blood loss (mL)	246.2 ± 12.3	101.2 ± 8.0	0.000
Postoperative drainage volume (mL)	187.9 ± 7.6	120.7 ± 6.4	0.000
Length of hospital stay (d)	9.0 ± 0.9	7.0 ± 0.9	0.000

### 3.3 Follow-up and treatment efficacy

All patients underwent follow-up, with a mean follow-up duration of 12.7 ± 1.7 months. Furthermore, no surgery-related complications such as incision infection, nerve damage, or cerebrospinal fluid leakage were observed in either group. In both groups (Table 3), VAS scores and ODI measured at 7 days postoperatively and at the final follow-up were significantly lower than the preoperative values, with statistically significant differences (all *P* < 0.05). However, comparisons of the VAS scores and ODI between the two groups at 7 days postoperatively and at the final follow-up indicated no statistically significant differences (all *P* > 0.05). The fusion rate was 89.5% (34/38) in the traditional channel group and 93.3% (28/30) in the bilateral channel group, with no statistically significant difference in fusion rates between the two groups (t = 0.310, *P* > 0.05).

**Table 3:** Comparison of VAS and ODI scores between traditional TLIF group and bilateral channel MIS-TLIF group.

Variables	Traditional (*n* = 38)	Bilateral channel (*n* = 30)	*P*
VAS for leg pain			
Preoperative	7.7 ± 0.7	7.5 ± 0.7	0.325
1 week postsurgery	3.5 ± 0.5^a^	3.3 ± 0.5^a^	0.079
Last follow-up	1.6 ± 0.5^a^	1.5 ± 0.6^a^	0.688
ODI (%)			
Preoperative	57.3 ± 3.2	57.5 ± 2.8	0.804
1 week postsurgery	34.4 ± 1.1^a^	33.9 ± 1.3^a^	0.078
Last follow-up	22.3 ± 2.8^a^	22.2 ± 2.5^a^	0.817

^a^ Is significant differences in *p*-value(*p* < 0.05) between the preoperative measurement and the other measurement.

## 4 Discussion

### 4.1 Advantages of bilateral channel technique in MIS-TLIF surgery

The MIS-TLIF approach contributes to a prolonged operative duration and increased radiation exposure due to fluoroscopy. Lian et al. (8) performed MIS-TLIF utilizing comprehensive 3D navigation, necessitating an average of 2.3 three-dimensional scans per procedure, with each patient receiving a mean radiation dose of 5.47 mSv. However, the operative duration was markedly extended, with single-segment procedures averaging 175.6 min and double-segment procedures requiring 270.2 min. Biase et al. (9) employed robot-assisted screw placement, reporting an average intraoperative radiation dose of 31.5 mSv per patient, which was considerably lower than the 59.5 mSv dose associated with x-ray fluoroscopy-assisted screw placement. Nonetheless, the average operative duration for single-segment procedures remained prolonged at 241 min. The findings of these studies indicate that various auxiliary techniques can effectively reduce the intraoperative radiation exposure. However, they concurrently contribute to a substantial increase in the operative duration. Furthermore, mastering these advanced navigation and robotic technologies requires a learning curve, and their intraoperative implementation further extends procedure time. Consequently, these methods fail to simultaneously address the dual objectives of minimizing both the operative duration and intraoperative radiation exposure. Klingler et al. (10) found that the use of 3D navigation reduces the radiation exposure of healthcare workers, but increases the radiation exposure of patients. It is believed that MIS-TLIF surgery should still be performed using two-dimensional x-ray fluoroscopy. The bilateral channel technique proposed in this study requires only an additional set of standard “five-piece set” instruments in conjunction with existing equipment, enabling simultaneous direct visualization for bilateral screw placement. This “five-piece set” comprises conventional retractors that are individually sterilized and packaged, thereby eliminating the additional costs and time associated with preparing an entirely new instrument set. In this study, applying the bilateral channel technique during MIS-TLIF procedures resulted in a significantly lower mean number of intraoperative fluoroscopic exposures in the bilateral channel group compared to the traditional TLIF group (9.5 vs. 13.9 exposures). Additionally, a comparative analysis of operative duration demonstrated that the mean operative time in the bilateral channel group was notably shorter than that in the traditional TLIF group (89.2 min vs. 120.0 min). Lee et al. (11) suggested that the prolonged operative duration in MIS-TLIF is attributable to the restricted surgical field of vision imposed by the channel, leading to an extended learning curve. Although the operative time decreases as the surgeon's experience improves, the requirement for alternating bilateral screw placement during the procedure continues to prolong the duration compared to open surgery. Direct visualization through bilateral channels is achieved by employing the bilateral channel technique, obviating the necessity of familiarizing oneself with additional auxiliary instruments, thereby avoiding time delays associated with a new learning curve.

In the present study, the bilateral channel group exhibited significantly lower intraoperative blood loss, postoperative drainage volume, and length of hospital stay than the traditional surgery group, with all differences reaching statistical significance. The mean difference in intraoperative blood loss between the groups exceeded 100 mL. Although the mean postoperative drainage volume differed by less than 100 mL, this parameter was found to influence the length of hospital stay and postoperative recovery, with the bilateral channel group experiencing a hospital stay that was, on average, two days shorter than that of the traditional surgery group.

### 4.2 Precautions for bilateral channel technique

The MIS-TLIF approach has been used for over 20 years and can reduce intraoperative bleeding, paraspinal muscle injury, and incision infection through minimally invasive techniques (12, 13). The bilateral channel technique for MIS-TLIF proposed in this study is primarily distinguished by simultaneous placement of bilateral screws. To optimize clinical outcomes, it is advisable that two surgeons proficient in this technique collaboratively perform the procedure. When feasible, dual electrocautery devices, dual suction systems, or even dual light sources can facilitate intraoperative handling. Electrocautery and suction devices must be alternated in the absence of these resources. On the decompression side, following the insertion of the fusion device, intact gelatin sponges should be temporarily packed into the intervertebral space to prevent bleeding from interfering with screw placement on both sides. These sponges can be removed once screw fixation is completed. Because the light source consists of dual-head optical fibers, allocating one for each side is sufficient to meet the illumination requirements for screw placement. During preoperative incision planning, markings are typically placed bilaterally along the midline, at the level of the targeted spinous process. However, because of the simultaneous screw placement involved in the bilateral channel technique, once the channel is positioned on the decompression side, the back skin shifts toward that side. This displacement occurs because during decompression and screw placement, an inclined angle is necessary, causing the upper end of the channel to exert habitual outward pressure, thereby shifting the skin toward the decompression side. Consequently, for the non-decompression side incision, it is advisable to position the incision 1 cm further laterally or extend the preoperative marking outward by approximately 1 cm. This issue is specific to simultaneous bilateral channel placement, whereas in conventional approaches, in which procedures on the decompression side are completed before removing the channel and creating an incision on the non-decompression side, such concerns are avoided. Regarding the timing of the incision on the non-decompression side is generally performed during bone grafting on the decompression side. A premature incision may interfere with procedures on the decompression side, whereas a delayed incision fails to take advantage of the benefits of simultaneous bilateral screw placement. Once screw placement on the decompression side is completed, the channel should be retracted to prevent prolonged stretching and compression of the skin, which can result in skin necrosis and impede wound healing. Additionally, an essential consideration is that while simultaneous screw placement is feasible under favorable conditions, in cases where challenges such as excessive bleeding occur on one side, prioritization should be established by first achieving hemostasis before proceeding with simultaneous bilateral screw placement once the condition has stabilized.

### 4.3 Clinical efficacy of bilateral channel technique

Previous studies have reported no statistically significant differences in postoperative VAS and ODI scores between open surgery and MIS-TLIF (14–17). This study found that both groups of patients had a decrease in VAS and ODI scores after surgery, especially on the 7th day after surgery. The mean VAS and ODI scores in the bilateral channel group were lower than those in the traditional posterior approach group, but the differences were not statistically significant. In addition, the follow-up period of this study was only 12.7 months, and based on short-term efficacy analysis, the bilateral channel MIS-TLIF technique can achieve recovery outcomes comparable to those of the traditional MIS-TLIF technique. Further research is needed to determine the long-term efficacy of bilateral channel MIS-TLIF. This comparative analysis revealed that the interbody fusion rate in the bilateral channel group was higher than that in the traditional posterior-approach group. However, the difference did not reach statistical significance, which aligns with the previously reported findings (18–20). Incomplete fusion was observed in both the groups. However, no indications of screw loosening, breakage, or exacerbation of lower back pain were noted. This outcome may be attributed to the relatively short follow-up period, which warrants further longitudinal observations. Surgical complications are closely related to the learning curve. Notably, no surgical complications were recorded in either group, likely due to the surgeon's proficiency in the procedures.

## 5 Limitations

The present study had some limitations. This was a single-center retrospective analysis, and the retrospective bias may have influenced the findings. Furthermore, the relatively small sample size necessitates further validation in multi-center studies with larger cohorts. In addition, this study lacks mid-to long-term efficacy evaluation, which needs to be analyzed in subsequent studies.

## 6 Conclusions

Bilateral channel MIS-TLIF is a feasible surgical procedure and it can reduce the surgical duration and radiation exposure associated with intraoperative fluoroscopy.

## Data Availability

The raw data supporting the conclusions of this article will be made available by the authors, without undue reservation.
